# Prospective assessment of pre-existing and *de novo* anti-HLA IgE in kidney, liver, lung and heart transplantation

**DOI:** 10.3389/fimmu.2023.1179036

**Published:** 2023-09-05

**Authors:** Jasmin Mucha, Ara Cho, Anna Marianne Weijler, Moritz Muckenhuber, Amun Georg Hofmann, Markus Wahrmann, Andreas Heinzel, Birgit Linhart, Pia Gattinger, Rudolf Valenta, Gabriela Berlakovich, Andreas Zuckermann, Peter Jaksch, Rainer Oberbauer, Thomas Wekerle

**Affiliations:** ^1^ Department of General Surgery, Division of Transplantation, Medical University of Vienna, Vienna, Austria; ^2^ Department of Dermatology, Medical University of Vienna, Vienna, Austria; ^3^ Department of Internal Medicine III, Division of Nephrology and Dialysis, Medical University of Vienna, Vienna, Austria; ^4^ Division of Immunopathology, Department of Pathophysiology and Allergy Research, Center for Pathophysiology, Infectiology and Immunology, Medical University of Vienna, Vienna, Austria; ^5^ Karl Landsteiner University of Health Sciences, Krems, Austria; ^6^ Department of Cardiac Surgery, Medical University of Vienna, Vienna, Austria; ^7^ Department of Thoracic Surgery, Medical University of Vienna, Vienna, Austria

**Keywords:** organ transplantation, kidney transplantation, clinical, immunology, donor-specific antibodies (DSA), antibody-mediated rejection, IgE

## Abstract

**Introduction:**

Antibody mediated rejection (ABMR) is a major factor limiting outcome after organ transplantation. Anti-HLA donor-specific antibodies (DSA) of the IgG isotype are mainly responsible for ABMR. Recently DSA of the IgE isotype were demonstrated in murine models as well as in a small cohort of sensitized transplant recipients. In the present study, we aimed to determine the frequency of pre-existing and *de novo* anti-HLA IgE antibodies in a cohort of 105 solid organ transplant recipients.

**Methods:**

We prospectively measured anti-HLA IgE antibodies in a cohort of kidney (n=60), liver, heart and lung (n=15 each) transplant recipients before and within one-year after transplantation, employing a single-antigen bead assay for HLA class I and class II antigens. Functional activity of anti-HLA IgE antibodies was assessed by an *in vitro* mediator release assay. Antibodies of the IgG1-4 subclasses and Th1 and Th2 cytokines were measured in anti-HLA IgE positive patients.

**Results:**

Pre-existing anti-HLA IgE antibodies were detected in 10% of renal recipients (including 3.3% IgE-DSA) and in 4.4% of non-renal solid organ transplant recipients (heart, liver and lung cohort). Anti-HLA IgE occurred only in patients that were positive for anti-HLA IgG, and most IgE positive patients had had a previous transplant. Only a small fraction of patients developed *de novo* anti-HLA IgE antibodies (1.7% of kidney recipients and 4.4% of non-renal recipients), whereas no *de novo* IgE-DSA was detected. IgG subclass antibodies showed a distinct pattern in patients who were positive for anti-HLA IgE. Moreover, patients with anti-HLA IgE showed elevated Th2 and also Th1 cytokine levels. Serum from IgE positive recipients led to degranulation of basophils *in vitro*, demonstrating functionality of anti-HLA IgE.

**Discussion:**

These data demonstrate that anti-HLA IgE antibodies occur at low frequency in kidney, liver, heart and lung transplant recipients. Anti-HLA IgE development is associated with sensitization at the IgG level, in particular through previous transplants and distinct IgG subclasses. Taken together, HLA specific IgE sensitization is a new phenomenon in solid organ transplant recipients whose potential relevance for allograft injury requires further investigation.

## 1 Introduction

Organ transplantation is a lifesaving treatment option to replace terminally failing organs. Over the last decades, the short-term outcome after solid organ transplantation was significantly improved due to progress in immunosuppression, medical and surgical management and donor/recipient matching. However, there is still an unmet need to extend long-term graft survival (1, 2). Antibody-mediated rejection (ABMR), elicited by donor-specific antibodies (DSA) directed against donor HLA-antigens is a leading cause of late graft loss. DSA can either pre-exist or develop *de novo* in patients after transplantation and are an important predictor of graft survival (3–8). Pre-existing DSA can cause hyper acute and early acute ABMR and can also lead to graft loss, whereas *de novo* DSA are mainly responsible for chronic rejection, which remains difficult to treat (9–11). Consequently, it has become state of the art to screen routinely for anti-HLA antibodies before and after transplantation. Clinical routine laboratories are focusing on the isotype IgG, with only a few studies having investigated IgM and IgA as well (12, 13). In addition, the clinical relevance of IgG subclasses in humoral rejection has been recognized over the last years. IgG1 and IgG3 antibodies can effectively bind and activate complement and further initiate inflammatory processes. IgG2 binds complement weakly and therefore triggers less inflammation than IgG1 or IgG3. The subclass IgG4 is not able to activate complement and is mainly seen upon chronic antigen exposure, e.g. after persistent contact to allergens or parasites. Moreover, IgG4 seems to prevent complement activation and to limit inflammatory response by competing with other subclasses for antigen binding (14). High serum levels of *de novo* IgG3-DSA were associated with the occurrence and increased severity of ABMR, while other publications suggested an association of IgG4-DSA and graft failure (15, 16). However, the role of distinct IgG subclasses needs to be further investigated for a better understanding of the humoral immunity and to better predict allograft survival.

The main functions of IgE antibodies include mediation of type I allergy and protection against helminth infections. IgE is the least abundant immunoglobulin isotype in the blood and is mainly found in tissue. It is found in ≈10,000-fold lower concentrations than IgG in peripheral blood. The half-life of free IgE is 2-3 days, but it gets stabilized when bound to effector cells (mast cells and basophils) (17). The origin of IgE production sites are still under investigation. IgE production was observed in peripheral blood and in various tissues, such as nasal mucosa and bone marrow (18). The Th2 cytokines IL-4 and IL-13 are inducing the immunoglobulin subclass switch to IgE production and are key cytokines in the allergic reaction. Binding of IgE to the high affinity receptor FcϵRI allows crosslinking of IgE-FcϵRI complexes upon antigen contact and leads to immediate mast cell and basophil degranulation and the release of various lipid mediators and vasoactive amines and chemokines. The low affinity receptor FcϵRII (CD23) is mainly located on the surface of B cells and dendritic cells which can activate CD4+ T cells by IgE-facilitated antigen presentation and contribute to the late phase allergic reaction (19). Recently, IgE autoantibodies have been observed in several autoimmune diseases. Autoreactive IgE antibodies were shown to promote IFN-α production by plasmacytoid dendritic cells, contributing to the progression of systemic lupus erythematosus (SLE). There is also evidence of intragraft deposition of anti-DNA IgE antibody in kidney tissues from patients suffering from lupus nephritis. Furthermore, elevated levels of IgE antibodies were suggested to contribute to atherosclerosis (20–23). Moreover, it was recently shown, that IgE might mediate a unique tumor protective immune response (24). Augmented IgE levels during chronic epithelial tissue inflammation, in contrast, were reported to promote epidermal cell growth which may lead to carcinogenesis (25).

The first evidence for the existence of IgE in kidney transplantation was published in 1979. The authors found increased amounts of IgE bound to basophils of kidney transplant patients and observed basophil degranulation upon stimulation with donor lymphocytes, but without directly identifying the responsible donor antigens (26). More recently, our group demonstrated the occurrence of IgE directed against donor HLA/MHC antigens in a retrospective cohort of highly sensitized kidney recipients and in several murine transplantation models using skin and cardiac allografts (27). Evidence for circulating anti-HLA specific IgE antibodies in renal transplant recipients with ABMR was more recently also demonstrated by another publication. Kidney patients with positive anti-HLA IgE antibodies also showed increased peripheral basophil levels. During acute ABMR the intragraft deposition of IgE, mast cells and activated basophils was demonstrated (28).

The prospective pilot study presented herein is exploratory in nature. We aimed to determine the incidence of pre-existing and *de novo* anti-HLA IgE antibodies, including IgE-DSA, in a cohort of kidney, liver, heart and lung transplant recipients. Additionally, we assessed whether the existence of IgE antibodies is associated with the development of specific IgG1-4 subclass antibodies. Functionality of IgE antibodies was determined by means of an *in vitro* basophil degranulation assay and Th1 and Th2 signature cytokines were measured.

## 2 Materials and methods

### 2.1 Study participants and design

This study is an investigator-initiated, prospective, single-center study. Participants were recruited at the Medical University of Vienna/Vienna General Hospital (AKH). The study was approved by the ethics review board of the Medical University of Vienna (EK no. 267/2011 and EK no. 1535/2016). 105 patients were enrolled upon written informed consent including, 60 kidney and 45 lung, heart and liver transplant recipients (15 for each organ cohort) and 20 healthy male volunteers. Blood was collected at baseline (pre-transplant), three months and twelve months post-transplant.

### 2.2 Processing of human serum samples

Blood samples were collected in a native blood vacutainer containing clot activator. After approx. 30 min of storage at room temperature (RT), the tube was centrifuged for 10 min at 1,500x g, at 10°C. The serum was aliquoted and stored at -80°C. Biomaterial was processed and stored according to standard operating procedures by the MedUni Wien Biobank in an ISO 9001:2015-certified environment (29).

### 2.3 Single antigen bead-based immunoassay for HLA-specific IgE measurement

To investigate HLA-specific IgE, a protocol for measuring HLA-specific IgG was adapted by our research group for this purpose (30). Briefly, LABScreen Single Antigen Bead HLA class I (#LS1A04) and HLA class II (#LS2A01) detection tests were purchased from One Lambda. For prevention of complement interference phenomenon, human serum was first adjusted to 10 mM EDTA (Invitrogen) (31, 32). In the next step, 6 µL serum and 1.5 µL of antigen coated beads were incubated in a V-bottom plate (Merck, Microplate Devices Uniplate) for 30 min on a shaker at 550 RPM at RT in the dark. Beads were washed five times for 7 min at 1,800x g at 4°C. Next, 25µg/mL of PE-conjugated anti-human IgE monoclonal antibody (Biolegend, clone MHE-18) was added to the beads and incubated for 30 min on a shaker at 550 RPM at RT in the dark. After incubation, beads were washed twice and resuspended in 55 µL buffer. Measurement of HLA-specific IgE was performed on the *Luminex Flow Analyzer 200*. Threshold definition was informed by several factors and calculated by using the mean of the median fluorescence intensity (MFI) values of six healthy volunteers plus 2* standard deviation (SD). The minimum cut-off value for a positive IgE signal was set at an MFI > 25 [based on our previous observations (27)], seen in Equation 1.

Equation 1: Definition for a positive HLA-specific IgE signal detected by Luminex based single-antigen bead immunoassay. Mean of the median fluorescence intensity (MFI) of six healthy donors (HD) plus two times the standard deviation (SD). As an additional condition, the following must also apply: HLA-specific IgE MFI > 25.


Treshold = Mean MFI of 6 HD*2SD

Cut−off for positive signal MFI > 25

### 2.4 Measurement of HLA-specific IgG subclasses

Human serum sample preparation was performed as described for HLA-specific IgE measurement. After incubation of serum and single-antigen beads (LABScreen Single Antigen Bead HLA class I and class II), samples were washed twice and incubated with PE-labeled monoclonal anti-human IgG subclass antibodies at concentrations of 12.5 µg/mL for IgG1, 10 µg/mL for IgG2, 16 µg/mL for IgG3 and IgG4 (Southern Biotech, IgG1 clone HP6001, IgG2 clone 31-7-4, IgG3 clone HP6050, IgG4 clone HP6025) for 30 min on a shaker at RT in the dark. After two washing steps, samples were measured on the *Luminex Flow Analyzer 200*. Threshold for positive signals was calculated by using Equation 1.

### 2.5 Measurement of Th1 and Th2 cytokine levels in sera from kidney recipients

Cytokine levels of IL-2, IL-4, IL-5, IL-6, IL-10, IL-13, INFγ and TNFα were investigated in serum samples from kidney transplant recipients. Samples were stained in technical duplicates according to the manufacturer’s protocol using the *LEGENDplex multi-analyte flow assay kit* (Biolegend) and subsequently measured with a *BD LSRFortessa* flow cytometer. Raw data were analyzed using the Qognit software (Biolegend Legendplex analysis software).

### 2.6 Functional *in vitro* mediator release assay with humanized rat basophil leukemia cells

RS-ATL8 cell line, a rat basophilic leukemia cell line expressing the human α, β, γ high affinity IgE receptor chain (huRBL) was kindly provided by Prof. Ryosuke Nakamura. The serum samples were pre-incubated overnight (o/n) in a 1:5 ratio in MEM medium, supplemented with 5% FCS, 1% L-Glutamin, 1% PenStrep, 0.25% Geneticin and 0.25% Hygromycin B. In a next step, 2x10^5^ huRBL cells were incubated together with sera from kidney transplant recipients or with HD serum overnight, to a final serum dilution of 1:10 and stimulated with HLA class II tetramers (DQA1*05:01, DQB1*02:01 and DQA1*01:01, DQB1*05:01, respectively; NIH Tetramer Core Facility). We used 5 µg/mL of DQA1*05:01, DQB1*02:01 and 0.12 µg/mL of DQA1*01:01, DQB1*05:01 tetramer. Mediator release of β-hexosaminidase was measured with a TECAN microplate reader at λ extinction: 360 nm and at λ emission: 465 nm. Mediator release was calculated by the β-hexosaminidase release after complete cell lysis (100%) by addition of 10% triton X-100. Samples were measured in triplicates, the background in six replicates.

### 2.7 Statistical analysis

Data were analyzed with GraphPad Prism8 (GraphPad Software, Inc) and IBM SPSS statistics version 25. Th1/Th2 cytokine values were analyzed as median with 95% confidence interval (CI). Values from huRBL assays were expressed as mean ± standard deviation (SD). P values were calculated using Fishers exact test and Mann-Whitney test, respectively, and p< 0.05 was considered as statistically significant.

## 3 Results

### 3.1 Frequency of pre-existing and *de novo* anti-HLA IgE antibodies in organ transplant recipients

**Figure 1:**
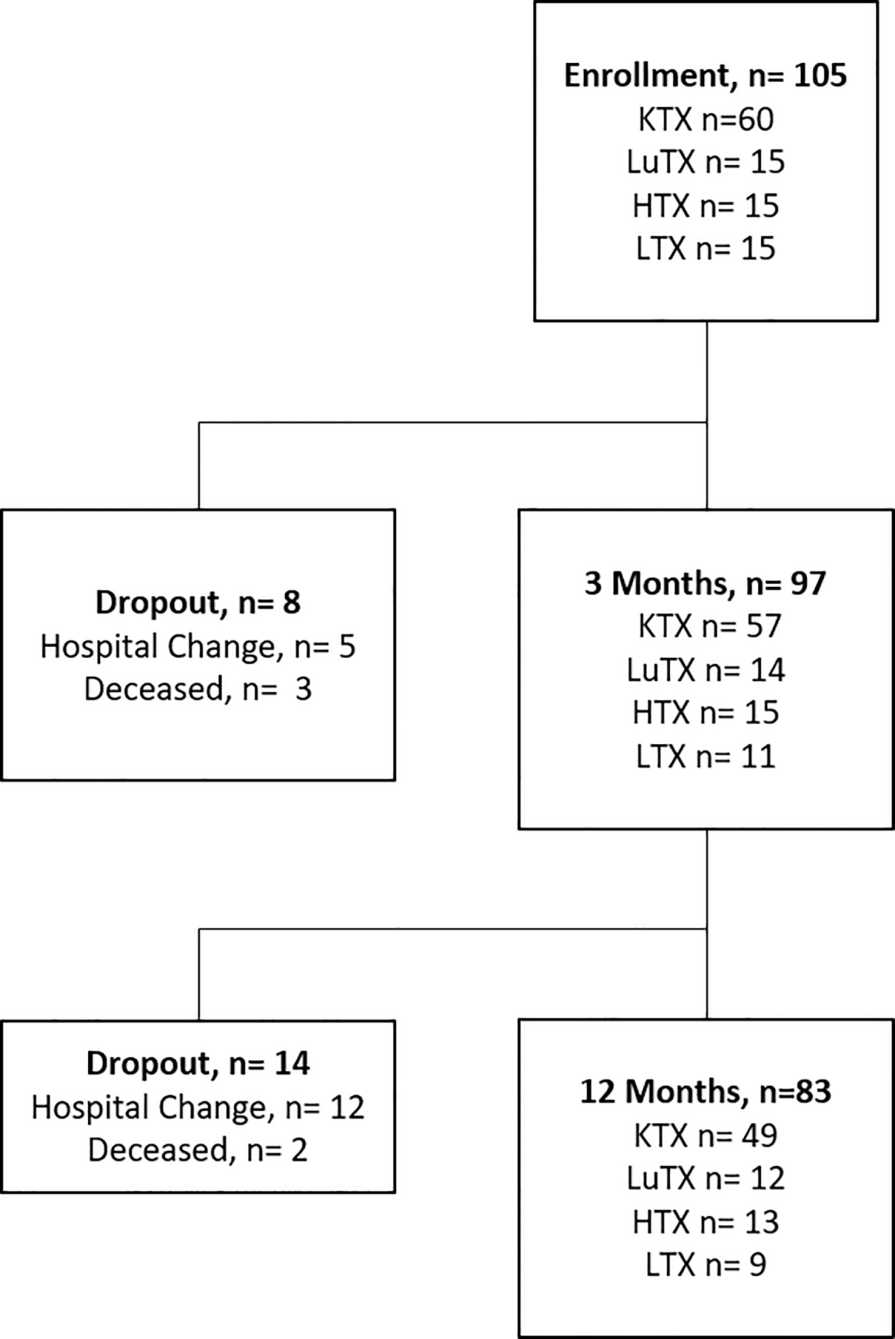
Study flowchart is illustrated. Study enrolment of 105 patients, including 60 kidney - (KTX), 15 lung- (LuTX), 15 heart - (HTX) and 15 liver (LTX) transplant recipients. In total, eight dropouts were registered after three months and 14 dropouts after 12 months. 83 transplant recipients finished the one-year study visit.

Sixty kidney (KTX), 15 lung (LuTX), 15 heart (HTX) and 15 liver (LTX) recipients were prospectively enrolled into the study before transplantation (**Figure 1**). Follow-up visits were performed at three- and 12-months post transplantation. Ninety-seven and 83 patients were available for analysis at three and 12 months respectively. Patient characteristics are listed in **Table 1**. The majority of patients were male (67.6%) with a mean age of 54.4 years at the time of transplantation. 16.7% of kidney transplant recipients received a re-transplant. Non-renal transplant recipients (lung, heart and liver) were analyzed as one cohort due to the lower sample size of the individual groups.

**Table 1:** Patient characteristics (anti-thymocyte globulin (ATG), extracorporeal photopheresis (ECP), immune adsorption (IAS)).

Recipient characteristics	All patients (n=105)	Kidney (n=60)	Heart, liver and lung (n=45)
Female**/**male, n (%)	34 (32.4)/ 71 (67.6)	19 (31.7)/ 41 (68.3)	16 (35.6)/ 29 (64.4)
Age, mean ± SD	54.4 ± 5.2	54.9 ± 12.7	54.2± 12.6
Living donor, n (%)	2 (1.9)	2 (3.3)	0 (0)
Previous transplants, n (%)	10 (9.5)	10 (16.7)	0 (0)
Immunosuppression			
Tacrolimus, n (%)	104 (99)	60 (100)	44 (97.8)
Cyclosporine, n (%)	1 (0.95)	0 (0)	1 (2.2)
Mycophenolate, n (%)	87 (82.9)	60 (100)	27 (60)
Corticosteroids, n (%)	105 (100)	60 (100)	45 (100)
Induction therapy (ATG**/**ECP**/**IAS Basiliximab**/**Alemtuzumab), n (%)	101 (96.2)	59 (98.3)	42 (93.3)
ATG, n (%)	28 (26.7)	1 (1.7)	27 (60)
ECP, n (%)	9 (8.6)	0 (0)	9 (20)
ATG+ECP combination, n (%)	1 (0.95)	0 (0)	1 (2.2)
IAS, n (%)	1 (0.95)	1 (1.7)	0 (0)
ATG+IAS, n (%)	5 (4.8)	5 (8.3)	0 (0)
Basiliximab, n (%)	52 (49.5)	52 (86.7)	0 (0)
Alemtuzumab, n (%)	5 (4.8)	0 (0)	5 (11.1)

**Figure 2:**
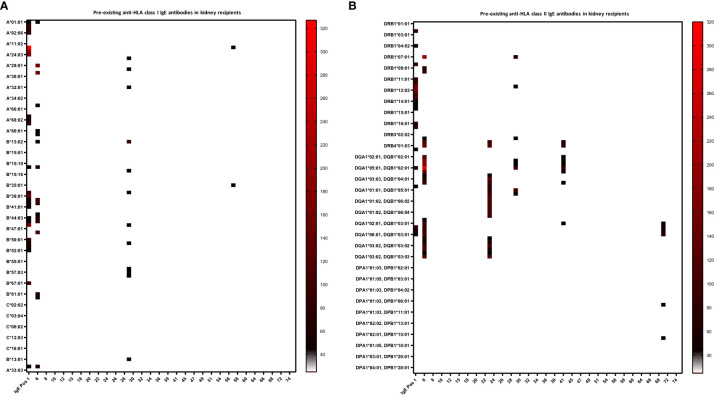
Heat-map of pre-existing anti-HLA class I and II IgE antibodies in kidney recipients. Baseline anti-HLA IgE levels (i.e. before transplant) are shown for the 60 kidney recipients. Serum of the kidney recipients and one IgE positive control (IgE Pos 1, a highly sensitized kidney transplant patient), were analyzed for anti-HLA IgE by a single-antigen bead-based Luminex assay. **(A)** Three kidney recipients were positive for IgE antibodies against HLA class I and **(B)** five patients against class II. MFI intensities are color coded from white (negative) to black (intermediated) and red (high). Threshold for positivity was calculated according to Equation 1, as mean MFI of six healthy donors (HD) + 2*SD and a cut-off MFI > 25. X-axis = patient ID, y-axis = HLA antigens (screening kit contained beads with 97 specificities for HLA class I and 95 for class II antigens).

Twenty percent of recipients had pre-existing anti-HLA IgG antibodies (16.7% kidney recipients and 24.4% non-renal recipients) and 11.4% showed pre-existing IgG-DSA (13.3% kidney recipients and 8.9% non-renal recipients) as detected in routine clinical diagnostic bead-based analysis (**Table 2**). IgE serum levels are drastically lower than IgG levels (x10,000), resulting in very low MFI signals in the Luminex-based bead assays. Therefore, we first defined a cut-off for IgE positivity. To this end, we screened 6 healthy male volunteers without any known sensitization events for anti-HLA IgE class I and II antibodies [males were selected since pregnancy can induce anti-HLA IgE (27)]. For each HLA antigen specificity, the threshold for positive MFI signals was calculated as mean MFI of 6 HD+ 2*SD (Equation 1). In addition, an MFI value > 25 was defined as minimum value required for positivity (27). Pre-existing anti-HLA IgE antibodies were found in 10% (6/60) of kidney recipients, and 4.4% (2/45) of non-renal transplant recipients (**Table 2**; **Figures 2A, B**). Three kidney recipients had anti-HLA class I and five patients HLA class II IgE antibodies before transplantation. Two of these patients demonstrated IgE against donor HLA (i.e. IgE-DSA). Moreover, one heart recipient was positive for anti-HLA class I IgE (not donor directed) and one liver patient for anti-HLA class II IgE antibodies (not donor directed) (no lung recipient was anti-HLA IgE positive) (**Supplementary Figures 1A, B**). All sensitized patients were polysensitized to more than one HLA antigen. All kidney recipients with pre-existing anti-HLA IgE antibodies also showed IgG antibodies and 83.3% (5/6) had also pre-existing IgG-DSA. Of those kidney recipients that had IgG-DSA, 25% also had IgE-DSA (2 of 8). IgE antibodies against specificities for which no IgG was detectable were evident in seven of eight patients. Vice versa, all eight recipients had IgG antibodies against specificities for which no IgE antibodies were detected.

**Table 2:** Pre-existing and *de novo* anti-HLA IgG and IgE.

	All patients (n=105)	Kidney (n=60)	Heart, liver and lung (n=45)
Pre-existing anti-HLA IgG, n (%)	21 (20)	10 (16.7)	11 (24.4)
Pre-existing IgG-DSA, n (%)	12 (11.4)	8 (13.3)	4 (8.9)
Pre-existing IgG-DSA class I/II, n (%)	5 (4.8)/ 7 (6.7)	2 (3.3)/ 6 (10)	3 (6.7)/ 1 (2.2)
*De novo* IgG-DSA, n (%)	14 (13.3)	6 (10)	8 (17.8)
Pre-existing anti-HLA IgE, n (%)	8 (7.6)	6 (10)	2 (4.4)
Pre-existing IgE-DSA, n (%)	2 (1.9)	2 (3.3)	0 (0)
*De novo* anti-HLA IgE, n (%)	3 (2.9)	1 (1.7)	2 (4.4)
*De novo* IgE-DSA, n (%)	0 (0)	0 (0)	0 (0)

**Figure 3:**
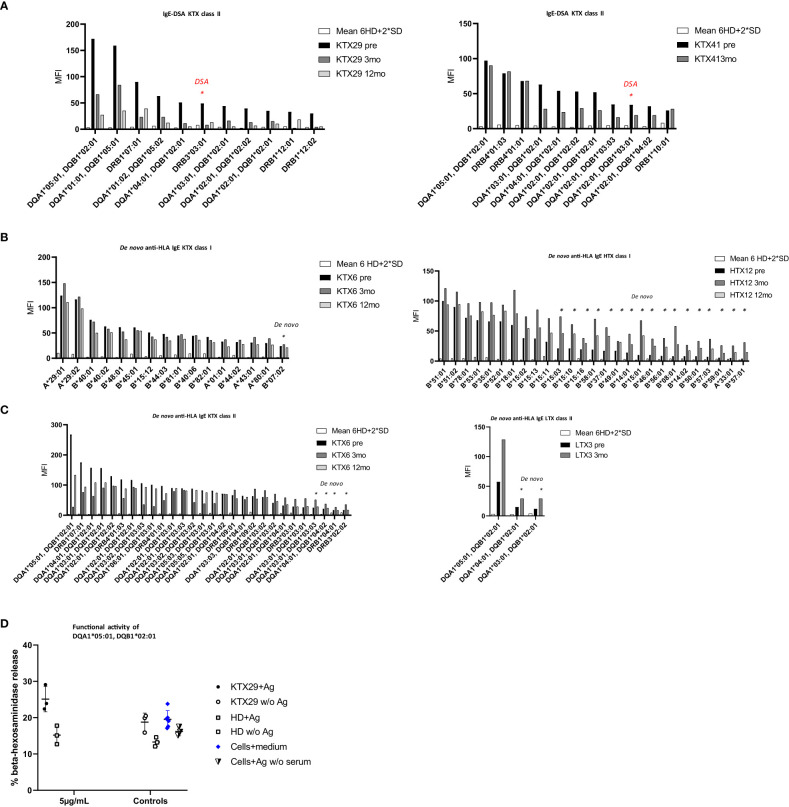
The occurrence of pre-existing, and *de novo* anti-HLA IgE antibodies and IgE-DSA in organ transplant patients. Anti-HLA IgE antibodies were measured at baseline (pre), three- (3mo) and 12 months (12mo) post-transplantation. **(A)** Anti-HLA IgE antibodies in the two kidney recipients showing pre-existing IgE-DSA: KTX29 (no. of positive reactivities pre: 11, 3mo: 2, 12mo: 3; IgE-DSA red indicated n= 1) and KTX41 (no. of positive reactivities pre: 11, 3mo: 7, IgE-DSA red indicated n= 1). **(B)** Anti-HLA IgE antibodies class I are shown from one kidney patient KTX6 (no. of positive reactivities pre: 15, 3mo: 16 including 1 *de novo* specificity, 12mo: 14) and one heart patient HTX12 (no. of positive reactivities pre: 10, 3mo: 27 including 17 *de novo* specificities, 12mo: 19). **(C)** Anti-HLA IgE antibodies class II from one kidney KTX 6 (no. of positive reactivities pre: 24, 3mo: 28 + 4 *de novo*, 12mo: 25) and one liver patient (no. of positive reactivities pre: 1, 3mo: 3 including *2 de novo* specificities) are depicted. X-axis = HLA antigen, y- axis = MFI signals. Threshold was calculated as mean MFI of 6 HD +2*SD and MFI > 25. **(D)** Functional activity of anti-HLA IgE antibody was assessed by using the selected antigen-tetramer DQA1*05:01, DQB1*02:01. Humanized rat basophil leukemia (huRBL) cells were tested for mediator release of β-hexosaminidase together with patient serum which was positive for the respective antigen (recipient KTX29 + Ag), a HD serum without detectable anti-HLA IgE antibody (HD + Ag) and cells together with serum was also tested without antigen (KTX29 w/o Ag, HD w/o Ag). Tetramer concentration of 5 µg/mL was used as indicated. huRBL cells were stimulated with antigen but without serum (Cells + Ag w/o serum) and background measurement was performed (cells + medium).

Pre-existing IgE-DSA were detectable in two kidney recipients (**Figure 3A**). One patient (KTX29) had the same IgE-DSA and IgG-DSA specificity against DRB3*03:01, whereas the other patient (KTX41) had IgE-DSA against DQA1*02:01, DQB1*03:01 without a corresponding IgG-DSA. Among the kidney re-transplant recipients, 70% were tested positive for anti-HLA IgG antibodies (versus 10% among primary transplant recipients; p< 0.001, Fishers exact test) and 40% had IgG-DSA. Of note, among the kidney re-transplant recipients 50% (5 of 10) were positive for pre-existing HLA-IgE antibodies (versus 2% among primary transplant recipients; p< 0.001, Fishers exact test) and 20% (2 of 10) had IgE-DSA. 83.3% of patients (5/6) with anti-HLA IgE antibodies were re-transplanted (three patients had their second, one had their third kidney transplant and one patient had a previous heart transplant). These findings suggest that patients receiving a re-transplant are more likely to develop anti-HLA IgE than patients receiving a first transplant. Three female transplant patients (1 kidney, 1 heart and 1 liver) already had pre-existing anti-HLA IgE without a previous transplantation. All three of them had given birth to at least one child. It has already been shown previously that anti-HLA IgE antibodies can develop as a consequence of pregnancy (27).

Next, we investigated the frequency of *de novo* HLA-specific IgE antibodies three and twelve-months post-transplantation. 1.7% of all kidney (1/60) and 4.4% of the non-renal transplant recipients (2/45) developed *de novo* anti-HLA IgE (**Figures 3B, C**) at low MFI signal intensities (MFI< 75). Patients who developed *de novo* anti-HLA IgE showed several pre-existing HLA specific IgG responses, including IgG-DSA. However, overall, we did not detect any *de novo* IgE-DSA in organ transplant recipients within one-year after transplantation. One kidney recipient with positive pre-transplant HLA-specific IgE antibodies developed *de novo* IgG-DSA but no IgE-DSA.

To investigate a possible relationship between atopy and anti-HLA IgE, we collected medical history records of transplant recipients. 63% of anti-HLA IgE positive patients had a history of atopy (5 of 8) versus 30% of anti-HLA IgE negative patients (29 of 97) (p= n.s.), showing a trend for a correlation of atopy with the occurrence of anti-HLA IgE. However, the low sample size of HLA-IgE positive patients was a limiting factor for our analysis.

Among the kidney transplant recipients positive for anti-HLA IgE antibodies, graft function was stable during the 12 month follow-up. At 3 months post-transplant, serum creatinine was 1.23 ± 0.46 mg/dL (mean ± SD) and GFR 50.6 ± 20.5 mL/min/1.73m^2^ (MDRD-IDMS formula, mean ± SD); at one-year post-transplant, creatinine was 1.07 ± 0.30 mg/dL (mean ± SD) and GFR 53.0 ± 18.4 mL/min/1.73m^2^ (MDRD-IDMS formula, mean ± SD). Among the liver and heart recipients who were positive for HLA-specific IgE, graft function also remained stable.

One lung and two kidney recipients (both negative for anti-HLA IgE) had a biopsy proven antibody-mediated rejection (ABMR) in the first-year post-transplant. Whereas, none of the anti-HLA IgE positive patients developed ABMR during the follow-up period. Nine kidney recipients experienced Banff borderline rejections, including one patient positive for anti-HLA IgE antibodies, and one had a suspected chronic rejection. 16.7% of anti-HLA IgE positive kidney recipients (1 of 6) experienced a borderline episode within the first-year post-transplant versus 14.8% of IgE negative patients (8 of 54) (p= n.s.). The sample size and the number of IgE positive recipients are limiting further conclusions about the potential impact of IgE antibodies on clinical graft outcomes.

The majority of anti-HLA IgE positive patients received induction therapy with anti-thymocyte globulin (ATG) (n= 6 out of 8), with three from those six patients receiving ATG receiving a re-transplant. Two IgE positive patients received ATG, three recipients a combination of ATG + immunoadsorption (IAS) therapy (kidney recipients), one patient received a combined ATG + ECP treatment (heart recipient) and two received the IL-2 receptor antagonist Basiliximab.

These data demonstrate that anti-HLA IgE antibodies and IgE-DSA occur in low frequency in renal and non-renal transplant recipients. The occurrence of anti-HLA IgE antibodies was accompanied in all patients by the presence of anti-HLA IgG antibodies, although the pattern of HLA-specificities was only overlapping but not identical in the majority of patients. Anti-HLA IgE is associated with sensitization through previous transplants or pregnancies.

### 3.2 Functional activity of anti-HLA IgE antibodies in kidney recipients

Functionality of anti-HLA IgE antibodies in kidney recipients was assessed by means of an *in vitro* basophil degranulation assay. Basophil mediator release assays are commonly performed in the field of IgE-mediated allergy (33). IgE antibodies bind to basophils via the IgE high affinity receptor FcϵRI and trigger degranulation upon cross-linking through recognition of the respective antigen. huRBL cells were incubated with serum from selected transplant patients and were stimulated with HLA-tetramers. The HLA antigen DQA1*05:01, DQB1*02:01 triggered degranulation in kidney recipient KTX29, who was sensitized against this antigen. Basophils showed higher mediator release by stimulation with the HLA-tetramer compared to the background (cells + medium) and to an unstimulated control (KTX29 w/o Ag) (**Figure 3D**). The % β-hexosaminidase release in the sensitized recipient KTX29 was 1.65-fold higher compared to the non-sensitized patient (HD). Similar results were observed with a second class II antigen (HLA-tetramer DQA1*01:01, DQB1*05:01) (**Supplementary Figure 1C**). These preliminary *in vitro* data suggest that anti-HLA IgE antibodies are functional and can mediate degranulation of effector cells.

### 3.3 Development of anti-HLA IgE antibodies is accompanied by Th1 and Th2 signature cytokines

**Figure 4:**
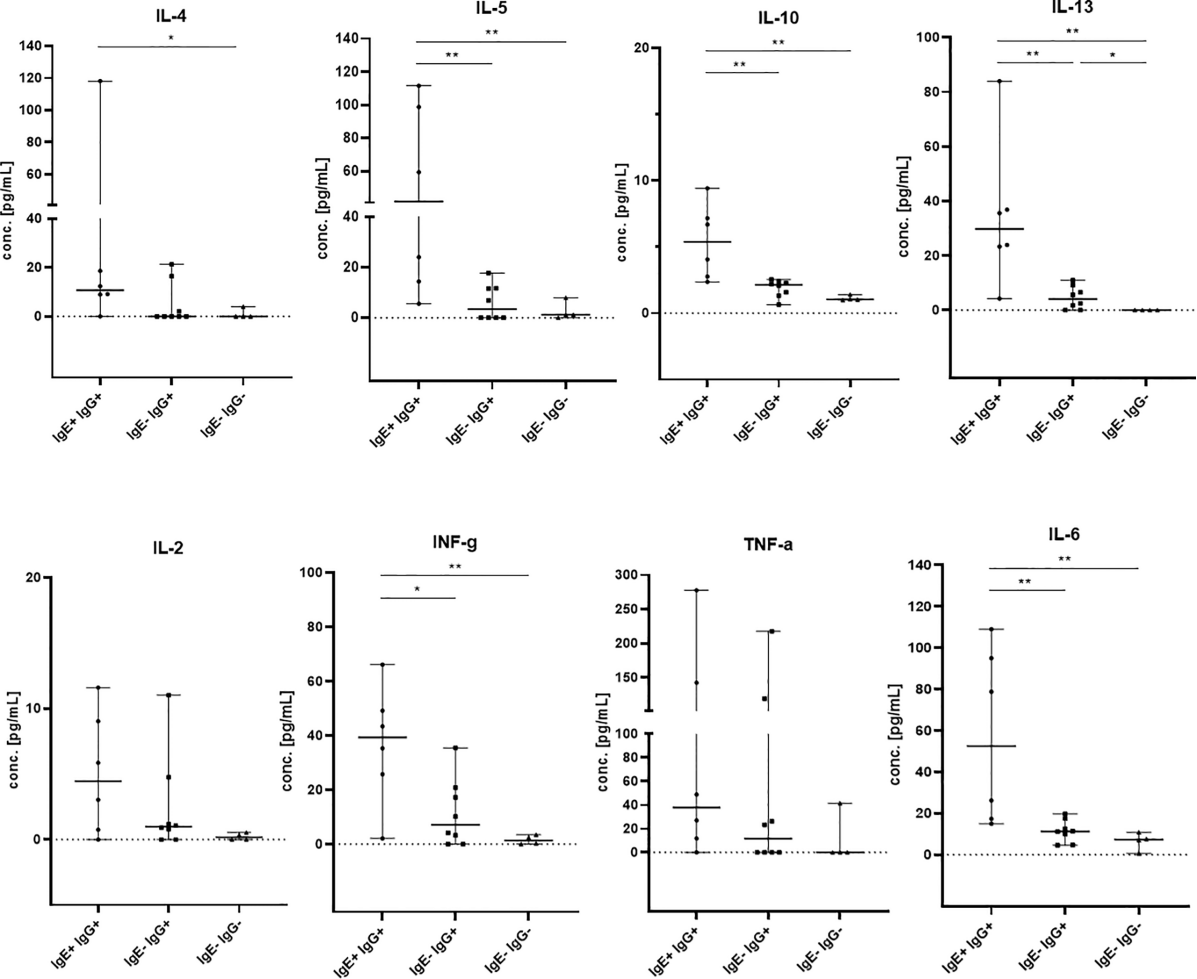
Th1 and Th2 cytokine levels in kidney transplant recipients. IL-2, IL-4, IL-5, IL-6, IL-10, IL-13, INFγ and TNFα were measured by flow cytometry in sera from patients which were positive for anti-HLA IgE antibodies (IgE+ IgG+, n= 6, baseline serum samples) and controls (IgE- IgG+, n= 8, 5 baseline and 3 samples after 12mo; IgE- IgG-, n= 4 baseline serum samples). The groups of patients are shown on the x-axis and cytokine concentrations are shown on the y-axis in pg/mL. Medians with 95% confidence interval (CI) are shown. Statistically significant differences between the groups are indicated as *p<0.05, **p<0.01.

IgE antibody production is part of the type 2 immune response and is stimulated by the Th2 cytokines IL-4 and IL-13. Therefore, we assessed Th1/Th2 serum cytokine levels by a bead-based multiplex assay (IL-2, IL-4, IL-5, IL-6, IL-10, IL-13, INFγ and TNFα) in all kidney recipients positive for anti-HLA IgE antibodies (IgE+IgG+, n=6) and in selected controls positive for anti-HLA IgG but negative for IgE (IgE-IgG+, n=8) and patients without any HLA-specific sensitization (n=4). Th2 cytokines were elevated in patients with anti-HLA IgE antibodies compared to controls negative for anti-HLA IgE and negative for anti-HLA IgG (IL-4: p<0.05; IL-5, IL-10, IL-13: p<0.01) and compared to controls negative for anti-HLA IgE but positive for anti-HLA IgG (p values calculated with the Mann-Whitney test) (**Figure 4**). Thus, the occurrence of anti-HLA IgE is associated with detectably higher serum Th2 cytokine levels. In addition, statistically higher levels were also observed for INFγ and IL-6 and a non-significant trend for elevated TNFα levels, suggesting an overall stronger immune response (including Th1) in IgE positive patients.

### 3.4 Anti-HLA IgE antibodies are associated with distinct IgG subclasses

**Figure 5:**
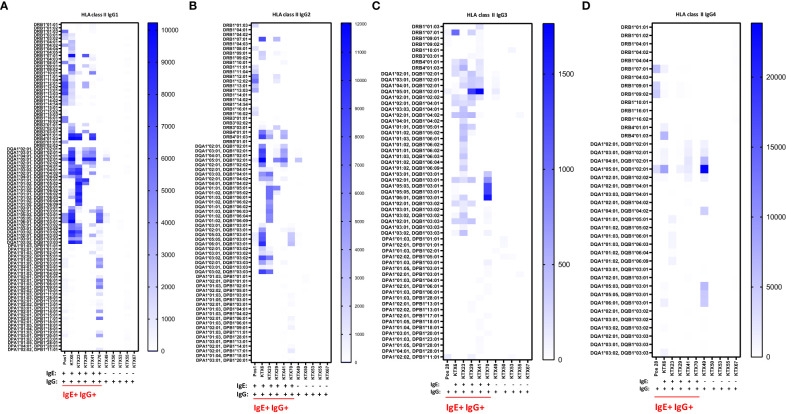
Serum levels of IgG subclasses 1-4 specific for HLA class II in kidney transplant recipients. Patients positive for anti-HLA IgE antibodies (IgE+ IgG+: KTX6, KTX23, KTX29, KTX41 and KTX70) and patients positive only for anti-HLA IgG antibodies (IgG+: KTX49, KTX50, KTX53, KTX55 and KTX67) were investigated for serum levels of **(A)** IgG1, **(B)** IgG2, **(C)** IgG3 and **(D)** IgG4 by single-antigen bead based assay for HLA class II and signal intensities were expressed as MFIs. Positive control serum (Pos 1) was used for investigation of IgG1 and IgG2 and control serum (Pos 28) for measurements of IgG3 and IgG4. Threshold for positivity was calculated as mean MFI of six HD + 2*SD with a minimum MFI > 25. IgE+ IgG+ patients are underlined in red. MFI intensities of bead signals for subclasses are color-coded in white (negative), light blue and dark blue. Only positive antigen reactivities, to which at least one patient from the measured cohort exhibited a positive signal, are depicted.

The IgG subclasses IgG1 and IgG3 have a high capability to bind complement and activating Fc receptors whereas antibodies of the IgG4 subclass do not. IgG2 binds weakly to complement. We measured IgG1-4 subclasses in IgE positive kidney recipients and in IgG positive but IgE negative patients and determined if a specific subclass is associated with the existence of anti-HLA IgE antibodies. In anti-HLA IgE positive patients (n=3 for class I, n=5 for class II), for each HLA antigen we assessed whether IgE occurred as a single isotype or together with IgG1, IgG2, IgG3 or IgG4 using a cross table. We found that IgE and IgG2 against the same HLA antigen were significantly more likely to occur together than individually (p<0.001, Fishers exact test). Associations between the development of anti-HLA IgE and other IgG subclasses (IgG1, IgG3 and IgG4) for the same specificity were also statistically significant, but to a lesser extent (using Cramer’s V as measure of correlation; class I: IgG2>IgG1>IgG4>IgG3; class II: IgG2>IgG3> IgG4>IgG1) (**Figures 5A-D** and **Supplementary Figures 2A-D**). Substantially higher IgG subclass antibody levels were observed among IgE positive recipients for all IgG subclasses, except IgG4 against HLA class II (comparing peak MFIs from IgE positive and IgE negative patients for each subclass, **Table 3**).

**Table 3:** Peak MFI signals of anti-HLA class I and II IgG subclass antibodies in IgE positive and IgE negative kidney transplant recipients.

HLA class I	IgG1	IgG2	IgG3	IgG4
IgE+ IgG+	6413 ± 738	6516 ± 2678	1430 ± 2196	4823 ± 7574
IgE- IgG+	197 ± 189	no positivity	46 ± 48	no positivity
HLA class II				
IgE+ IgG+	9003* ± 1192	7068* ± 3253	1272* ± 497	2747 ± 3499
IgE- IgG+	610 ± 1230	574 ± 1227	60 ± 49	4807 ± 10655

Means +/-SD of peak MFIs are shown. *p<0.01 (Mann-Whitney test).

Therefore, these data indicate that IgG subclass distribution is different in anti-HLA IgE positive patients.

## 4 Discussion

Late graft loss caused by antibody-mediated rejection (ABMR) is a major factor limiting long-term outcome after renal and non-renal transplantations (6, 34, 35). Despite some recent progress, ABMR remains difficult to treat (36). Donor-specific antibodies (DSA) directed against donor HLA antigens are instrumental in ABMR (37), with antibodies against non-HLA donor antigens also contributing (38, 39). The immunological mechanisms responsible for the pathology of ABMR remain incompletely understood and diagnostic challenges persist (40, 41). The recent discovery that IgE antibodies directed against donor HLA antigens occur in transplant recipients, adds a new piece to the ABMR puzzle (27). In the present study we systematically and prospectively assessed the frequency of pre-existing and *de novo* HLA-specific IgE antibodies in recipients of renal and non-renal transplants.

IgE has unique properties that sets it apart from all other antibody isotypes. The main site of action for IgE lies within tissues, where it persists bound to its main effector cells, mast cells and basophils. Upon binding of a multivalent antigen and cross-linking, IgE triggers effector cell activation through its FcεRI high affinity receptor. As a result, numerous pro-inflammatory events are initiated, including histamine and lipid mediator release and upregulation of cytokine production, including TNFα and IL-6. A second major effector pathway of IgE is mediated through its low affinity receptor FcεRII (CD23). CD23 positive B cells can bind soluble IgE-antigen complexes and were shown to thereby promote antigen presentation and the antigen-specific T cell response in allergy (42). Thus, IgE targeting HLA is hypothesized to act very differently from anti-HLA IgG.

Following a cohort of 60 renal and 45 non-renal (liver, lung and heart) transplant recipients, we found pre-existing anti-HLA IgE antibodies in 10% of kidney recipients and 4% of non-renal transplant recipients. Anti-HLA IgE was functional as it could trigger mediator release *in vitro* in a basophil degranulation assay. Our experimental set up was limited by the availability of the required HLA-tetramers in combination with the low number of anti-HLA IgE positive patients. Analyses of larger sample sizes in the future would provide additional insight into the functional activity of anti-HLA IgE. Anti-HLA IgE was found less frequently than anti-HLA IgG (17% in kidney recipients and 24% in non-renal solid organ transplant recipients), but was nevertheless present in a considerable number of patients. Anti-HLA IgE occurred only in patients that also had anti-HLA IgG and most of them even had IgG-DSA. These data could indicate that anti-HLA IgE develops predominantly through sequential class switch from anti-HLA IgG antibodies. IgE can derive from two pathways of development, the direct route (IgM → IgE) and the indirect/sequential route (IgM → IgG → IgE) (43). However, as some HLA specificities targeted by IgE but not by IgG were also observed in our cohort, direct class switch might occur for some anti-HLA IgE antibodies. Notably, pre-existing anti-HLA IgE was particularly frequent in re-transplant patients (50%). Remarkably, among the anti-HLA IgE positive patients (n=8), five had a previous transplant and three female recipients (kidney, heart and liver) without a previous transplant had at least one child (one had 5 children and one had 2). So either transplantation or pregnancy were identifiable sensitization events in all anti-HLA IgE positive patients. Patients with anti-HLA IgE showed not only evidence for a stronger Th2 response as assessed by cytokine serum levels, but also displayed higher levels of INFγ and IL-6, suggesting a stronger Th1 response as well. Collectively, these data suggest that the development of anti-HLA IgE is associated with the strength of the allo-response.

Pre-existing IgE-DSA were observed rarely (only in two patients) and these IgE-DSA became undetectable post-transplant. No *de novo* IgE-DSA became detectable within one-year post-transplantation. The sample size of the present study – even though being the largest cohort analyzed for anti-HLA IgE to date – limits a detailed analysis of IgE-DSA. Likewise, the potential effect of anti-HLA IgE on clinical endpoints cannot be assessed due to the size of the present cohort. 16.7% of anti-HLA IgE positive patients had a Banff borderline rejection episode, a similar frequency as IgE negative patients. ABMR was detected in three solid organ recipients, who were IgE negative. Long term data (beyond 1 year) of anti-HLA IgE positive patients could provide insight about the impact of IgE antibodies on the long-term allograft survival and if specific immunosuppressive regimens are required. However, larger sample sizes are required to definitively answer the question of the clinical impact of anti-HLA IgE. The current study is important in this respect as it demonstrates that anti-HLA IgE occurs only in IgG positive patients. Therefore, anti-HLA IgE studies in the future can focus only on pre-screened IgG positive patients, dramatically increasing the feasibility of large scale studies investigating IgE. While great care was taken in the establishment and execution of the assays measuring anti-HLA IgE in order to avoid false negative and false positive results (30), the minute quantities of IgE remain a methodological challenge.

A recent cross-sectional study investigating kidney recipients diagnosed with ABMR found circulating anti-HLA IgE (with the same specificities as IgG) and identified in the graft deposits of IgE and the presence of mast cells and activated basophils (28). Moreover, another recent paper revealed that elevated eosinophil counts were associated with an increased risk of subsequent rejection and a trend to a higher incidence of *de novo* (IgG) DSA (44). While IgE was not analyzed, eosinophils are another hallmark of type 2 inflammation, thus these data also indirectly point to a role for Th2-driven immunity, including IgE in alloreactivity.

In summary, IgE antibodies targeting HLA occur in renal and non-renal transplant recipients. Given the unique effector mechanisms of IgE the possible role and relevance of IgE-DSA in immune injury of allografts should be further investigated.

## Data availability statement

The original contributions presented in the study are included in the article/**Supplementary Material**. Further inquiries can be directed to the corresponding author.

## Ethics statement

The studies involving human participants were reviewed and approved by the ethics review board of the Medical University of Vienna. The patients/participants provided their written informed consent to participate in this study.

## Author contributions

JM and TW designed the research, interpreted data and wrote the manuscript. JM performed research. AC, AW, MM, AGH, GB, AZ, PJ and RO contributed to patient recruitment and sample collection and processing. JM, MW and TW analyzed the data. AH and MM contributed to statistical analysis. MW, BL, PG and RV contributed with analytical tools and reagents. All authors contributed to the article, reviewed the manuscript and approved the submitted version.

## Supplementary material

Supplementary Figure 1 Anti-HLA IgE antibodies against class I and II in kidney transplant recipients. Anti-HLA IgE class I antibodies were measured in serum of kidney recipient pre-transplantation, after three- and 12-months post-transplant, from **(A)** KTX29 (no. of positive reactivities pre: 13, 3mo: 2, 12mo: 2) and from KTX57 (no. of positive reactivities pre: 4, 3mo: 3, 12mo: 3). **(B)** HLA class II antibodies were measured in kidney patient serum from KTX23 (no. of positive reactivities pre: 20, 3mo: 20, 12mo: 20) and from KTX70 (no. of positive reactivities pre: 11). Threshold was calculated using the mean MFI of six HD + 2*SD, minimum cut-off MFI > 25. **(C)** Basophil mediator release assay was performed by using the HLA-tetramer DQA1*01:01, DQB1*05:01 together with serum from kidney patient KTX29, who was sensitized against that respective antigen (recipient KTX29 + Ag). As control measurement, huRBL cells were incubated with serum from a HD (HD + Ag) and serum without antigen (KTX29 w/o Ag, HD w/o Ag), cells were stimulated with respective antigen but without serum (cells + Ag w/o serum) and with medium (cells + medium). Tetramer was used at a concentration of 0.12 µg/mL, as indicated.

Supplementary Figure 2 Subclass analysis of IgG1-4 class I antibodies in kidney transplant recipients. Measurements of IgG subclasses of kidney patients with positive anti-HLA IgE (IgE+ IgG+: KTX6, KTX29 and KTX57) and HLA-specific IgG antibodies class I (IgG+: KTX25, KTX60 and KTX61) were performed and expression levels of **(A)** IgG1, **(B)** IgG2, **(C)** IgG3 and **(D)** IgG4 are shown. Positive control serum (Pos 1) was used for IgG1 and IgG2, and positive control serum (Pos 321) for IgG3 and IgG4 measurements. Threshold for positive MFI signals was calculated by using the mean MFI signal of six HD + 2*SD and the signal was required to exceed MFI > 25. The MFI signal intensities are color coded from white (negative), light blue (intermediate) and to dark blue (strong). Patients positive for IgE+ IgG+ antibodies are underlined in red.
