# Supplementary figures and images for: Prospective assessment of pre-existing and de novo anti-HLA IgE in kidney, liver, lung and heart transplantation
Source: Front Immunol. 2023 Sep 5;14:1179036. doi: 10.3389/fimmu.2023.1179036 (PMC10507692; doi:10.3389/fimmu.2023.1179036)

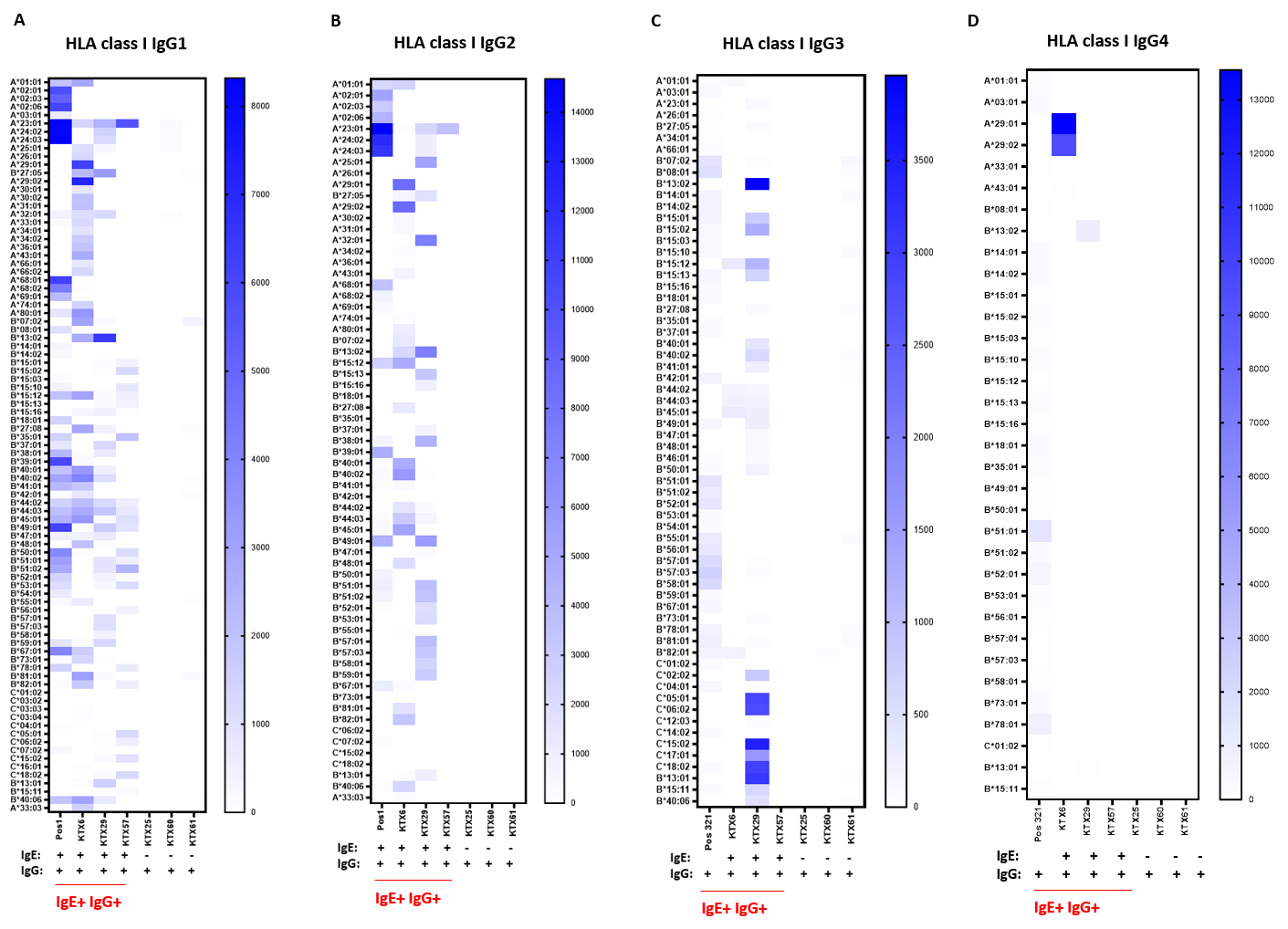

Supplement: Supplementary Figure 1 — Anti-HLA IgE antibodies against class I and II in kidney transplant recipients. Anti-HLA IgE class I antibodies were measured in serum of kidney recipient pre-transplantation, after three- and 12-months post-transplant, from (A) KTX29 (no. of positive reactivities pre: 13, 3mo: 2, 12mo: 2) and from KTX57 (no. of positive reactivities pre: 4, 3mo: 3, 12mo: 3). (B) HLA class II antibodies were measured in kidney patient serum from KTX23 (no. of positive reactivities pre: 20, 3mo: 20, 12mo: 20) and from KTX70 (no. of positive reactivities pre: 11). Threshold was calculated using the mean MFI of six HD + 2*SD, minimum cut-off MFI > 25. (C) Basophil mediator release assay was performed by using the HLA-tetramer DQA1*01:01, DQB1*05:01 together with serum from kidney patient KTX29, who was sensitized against that respective antigen (recipient KTX29 + Ag). As control measurement, huRBL cells were incubated with serum from a HD (HD + Ag) and serum without antigen (KTX29 w/o Ag, HD w/o Ag), cells were stimulated with respective antigen but without serum (cells + Ag w/o serum) and with medium (cells + medium). Tetramer was used at a concentration of 0.12 µg/mL, as indicated. [file DataSheet_1.zip › Suppl. Figure 2A-D.tif]

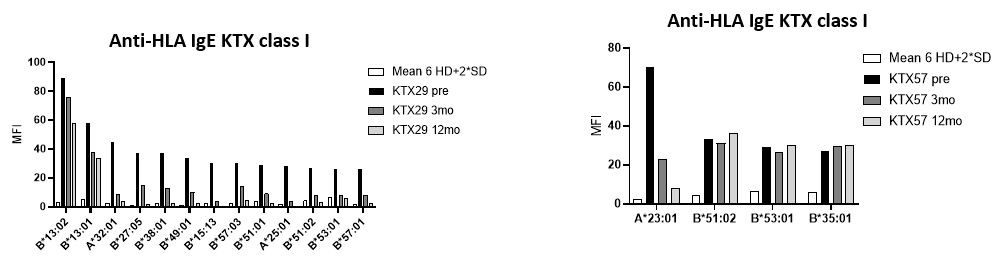

Supplement: Supplementary Figure 1 — Anti-HLA IgE antibodies against class I and II in kidney transplant recipients. Anti-HLA IgE class I antibodies were measured in serum of kidney recipient pre-transplantation, after three- and 12-months post-transplant, from (A) KTX29 (no. of positive reactivities pre: 13, 3mo: 2, 12mo: 2) and from KTX57 (no. of positive reactivities pre: 4, 3mo: 3, 12mo: 3). (B) HLA class II antibodies were measured in kidney patient serum from KTX23 (no. of positive reactivities pre: 20, 3mo: 20, 12mo: 20) and from KTX70 (no. of positive reactivities pre: 11). Threshold was calculated using the mean MFI of six HD + 2*SD, minimum cut-off MFI > 25. (C) Basophil mediator release assay was performed by using the HLA-tetramer DQA1*01:01, DQB1*05:01 together with serum from kidney patient KTX29, who was sensitized against that respective antigen (recipient KTX29 + Ag). As control measurement, huRBL cells were incubated with serum from a HD (HD + Ag) and serum without antigen (KTX29 w/o Ag, HD w/o Ag), cells were stimulated with respective antigen but without serum (cells + Ag w/o serum) and with medium (cells + medium). Tetramer was used at a concentration of 0.12 µg/mL, as indicated. [file DataSheet_1.zip › Suppl.Figure 1A.tif]

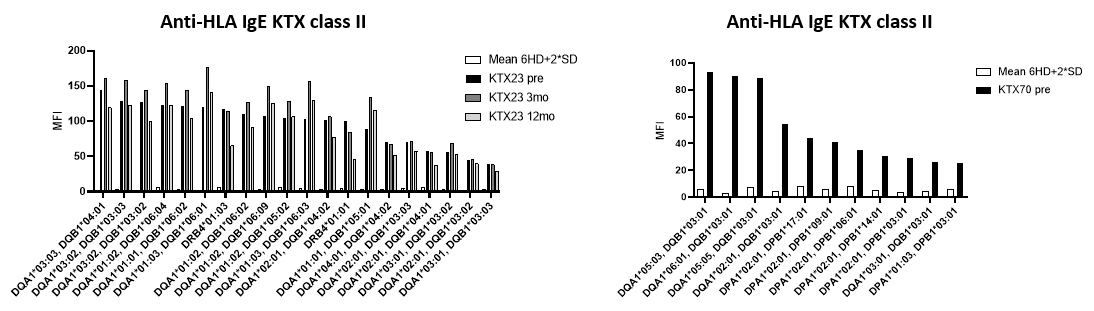

Supplement: Supplementary Figure 1 — Anti-HLA IgE antibodies against class I and II in kidney transplant recipients. Anti-HLA IgE class I antibodies were measured in serum of kidney recipient pre-transplantation, after three- and 12-months post-transplant, from (A) KTX29 (no. of positive reactivities pre: 13, 3mo: 2, 12mo: 2) and from KTX57 (no. of positive reactivities pre: 4, 3mo: 3, 12mo: 3). (B) HLA class II antibodies were measured in kidney patient serum from KTX23 (no. of positive reactivities pre: 20, 3mo: 20, 12mo: 20) and from KTX70 (no. of positive reactivities pre: 11). Threshold was calculated using the mean MFI of six HD + 2*SD, minimum cut-off MFI > 25. (C) Basophil mediator release assay was performed by using the HLA-tetramer DQA1*01:01, DQB1*05:01 together with serum from kidney patient KTX29, who was sensitized against that respective antigen (recipient KTX29 + Ag). As control measurement, huRBL cells were incubated with serum from a HD (HD + Ag) and serum without antigen (KTX29 w/o Ag, HD w/o Ag), cells were stimulated with respective antigen but without serum (cells + Ag w/o serum) and with medium (cells + medium). Tetramer was used at a concentration of 0.12 µg/mL, as indicated. [file DataSheet_1.zip › Suppl.Figure 1B.tif]

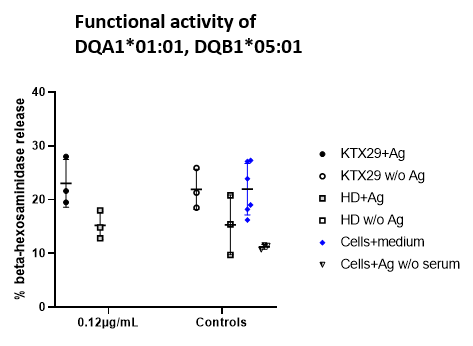

Supplement: Supplementary Figure 1 — Anti-HLA IgE antibodies against class I and II in kidney transplant recipients. Anti-HLA IgE class I antibodies were measured in serum of kidney recipient pre-transplantation, after three- and 12-months post-transplant, from (A) KTX29 (no. of positive reactivities pre: 13, 3mo: 2, 12mo: 2) and from KTX57 (no. of positive reactivities pre: 4, 3mo: 3, 12mo: 3). (B) HLA class II antibodies were measured in kidney patient serum from KTX23 (no. of positive reactivities pre: 20, 3mo: 20, 12mo: 20) and from KTX70 (no. of positive reactivities pre: 11). Threshold was calculated using the mean MFI of six HD + 2*SD, minimum cut-off MFI > 25. (C) Basophil mediator release assay was performed by using the HLA-tetramer DQA1*01:01, DQB1*05:01 together with serum from kidney patient KTX29, who was sensitized against that respective antigen (recipient KTX29 + Ag). As control measurement, huRBL cells were incubated with serum from a HD (HD + Ag) and serum without antigen (KTX29 w/o Ag, HD w/o Ag), cells were stimulated with respective antigen but without serum (cells + Ag w/o serum) and with medium (cells + medium). Tetramer was used at a concentration of 0.12 µg/mL, as indicated. [file DataSheet_1.zip › Suppl.Figure 1C.tif]
